# Surviving Fifty Years With Shrapnel Within the Brain

**DOI:** 10.1192/bjo.2022.357

**Published:** 2022-06-20

**Authors:** Muhammad Sayed Inam

**Affiliations:** Sylhet MAG Osmani Medical College, Sylhet, Bangladesh

## Abstract

**Aims:**

Traumatic brain injury during the war by shrapnel or bullet is lethal and life-threatening. The mortality rate from traumatic brain injury is more than 90%. Mrs. N (70) of Bangladesh is an exceptional and one of the luckiest women of the world. She is a war victim and has been living with shrapnel within her brain for the last 50 years.

**Methods:**

According to the patient attendant, half a century ago, during the Liberation War of Bangladesh, one shell accidentally exploded in their backyard. Unfortunately, a few pieces of shrapnel penetrated her arm, leg and right side of the head. She lost her consciousness and immediately treated at war hospital as per level best. She forgets details of her treatment and has no treatment records. Last 50 years, she has been surviving with movement difficulties, weaknesses of the upper and lower limbs and occasional convulsions. Her sufferings have intensified day by day. Last few years she has been experiencing headaches, dizziness, vomiting and forgetfulness. Two months back, she drank some insecticide mistakenly. She also suffering insomnia and she often cried out from deep sleep. She complains about hearing unknown voices. The voices were talking about her. She also started to suspect her family members. She claims that her family members were conspiring to kill her. She has history of convulsions three times in the last 50 years, which were generalized and tonic clonic in nature. She didn't take any medication for convulsion. As her suspicion, irrelevant talk and oddity in behavior worsened, her family members took her to me for treatment. Considering her past traumatic brain injury, an X-ray skull was advised. Surprisingly, it showed a metallic foreign body (shrapnel) within her skull. Furthermore, a CT scan of the brain was done, and it showed there was an irregular bony gap at right parietal area, a shrapnel at right frontal area with extensive encephalomalacic Porencephaly communicating with lateral ventricle. Her thyroid and liver function are normal. She is non-diabetic, non-hypertensive. She was advised to take risperidone 2 mg daily at night. With this low dose antipsychotic medication her psychosis controlled.

**Results:**

Surviving with shrapnel within the brain followed by traumatic brain injury is very much rare. Shrapnel gradually damages the brain parenchyma and creates a large porencephaly.

**Conclusion:**

Traumatic brain injury by bullet or shrapnel is always fatal. It is very much rare in medical to survive fifty years with shrapnel within the brain after shrapnel injury.

